# Fibrous Tumor of the Uterus Complicating Labor

**Published:** 1858-09

**Authors:** J. N. Kirkpatrick

**Affiliations:** Randolph County, Arkansas


*Fibrous Tumor of the Uterus complicating Labor.* Reported by J. N. KIRKPATRICK, M. D., of Randolph County, Arkansas.

Mrs. H—— was taken in labor on the 23d of December, 1857; the symptoms were those of ordinary labor, and the case progressed as common for a considerable length of time, when a large fleshy substance alarmed the midwife that was in attendance, and Dr. B. was sent for; the Doctor living some miles distant, a considerable time elapsed before he could get there, and prior to his arrival the child was born, the placenta expelled, &c. But this large fleshy substance was still there—hanging down between the woman's thighs; the doctor on examining the case, said that the womb was prolapsed and inverted, or, literally speaking, that it was inside out; he said, moreover that it was very much swollen, and that as soon as he could get the swelling reduced he would put it back, and that she would be well in a short time, &c. He continued his attendance on her for near two months, applying his washes and poultices to reduce the swelling of the womb, but without effect. Finally, the Doctor and friends gave her up to die, and after being left alone for two weeks, I was sent for to see her; on my arrival I found her presenting the following symptoms, viz: she was perfectly anæmic—not having the color of red blood about her—being very much emaciated, completely bed-ridden, with large bed sores on her hips and back; for in this situation she had been kept for the last two months—not being allowed to arise to obey any of the calls of nature; she did not however suffer much pain, except in her lower extremities, which were always cold and painful, especially towards evening.

At first I considered the case hopeless, and told her that I could do her no good; but she insisted that I should do something, saying, that she believed that I could cure her if I would try. On examining the case, I found the womb in its natural position, and that this fleshy substance (which had given rise to all the trouble) was a large polypus, that had come down soon after labor commenced. It was as large as a full sized cocoa-nut, and of a conical shape, and attached to the mouth of the womb by a narrow neck or pedicle, and hanging out by the side of the external organs of generation; with this diagnosis I immediately proceeded to remove it with the knife, by cutting or dividing the pedicle near its attachment, and removed the whole in one solid mass. It bled but very little, and weighed near three pounds. On opening the tumor I found it to be of a hard fleshy texture with two small cavities containing a small amount of fluid. I used an astringent wash per vaginum for a few days. Ordered a nourishing diet, with the muriated tincture of iron three times a day, the lower extremities to be frequently sponged with salt and water, and moderate exercise every day. Convalescence commenced at once and progressed rapidly, and she is now well.

This case clearly shows the necessity of a thorough examination in every case, as well as the impropriety of abandoning a patient however hopeless.—*St. Louis Med. & Surg. Journal.*

